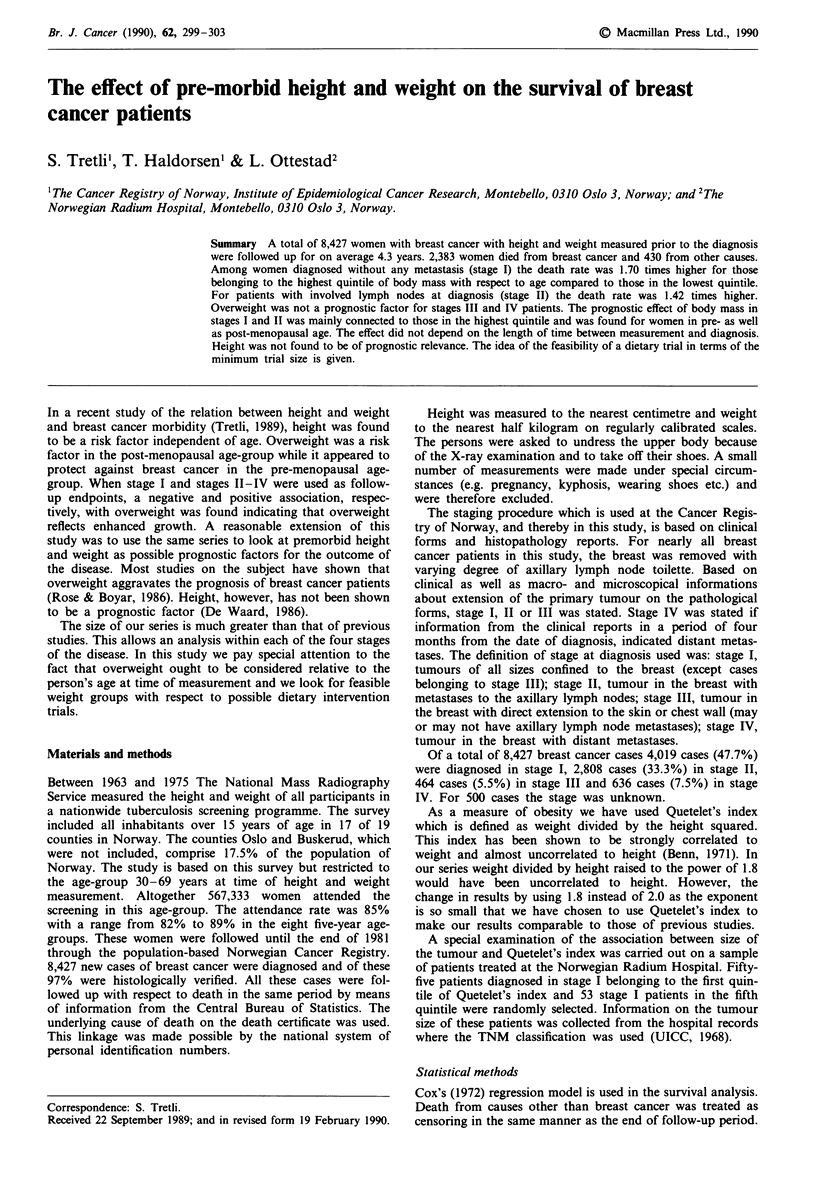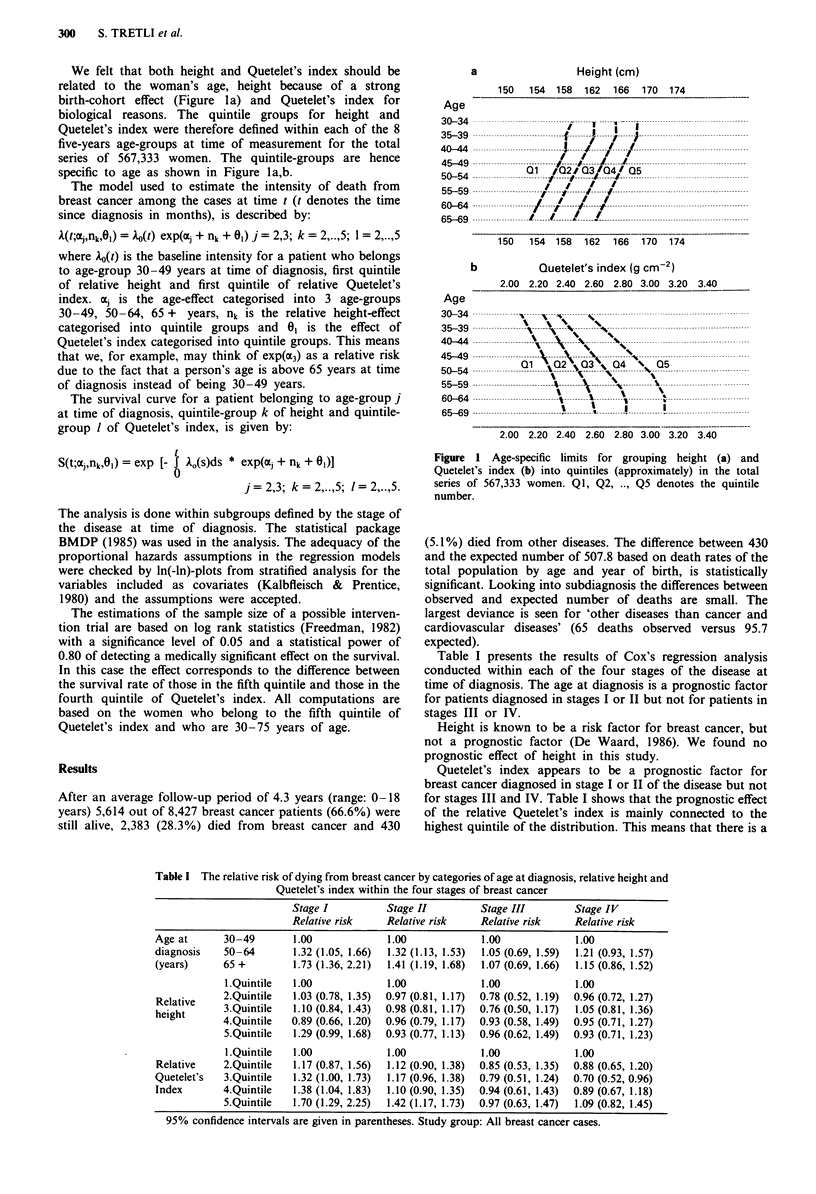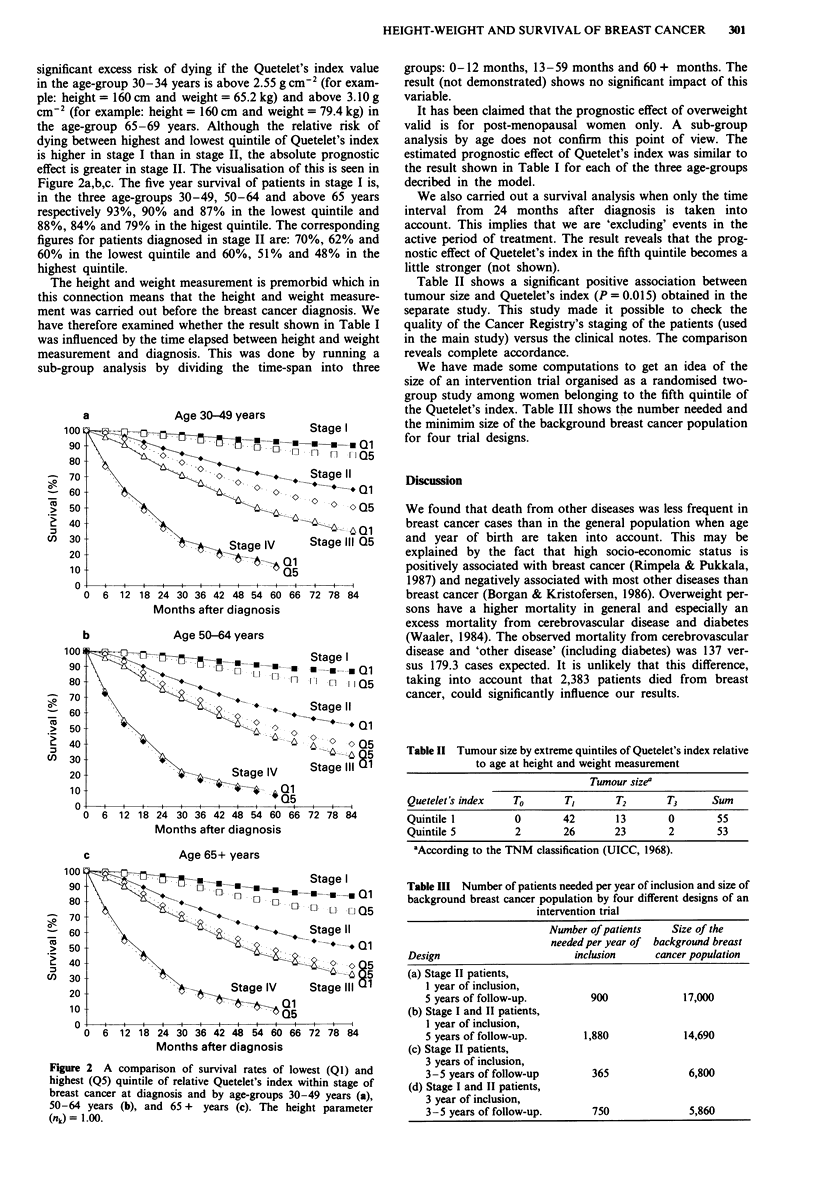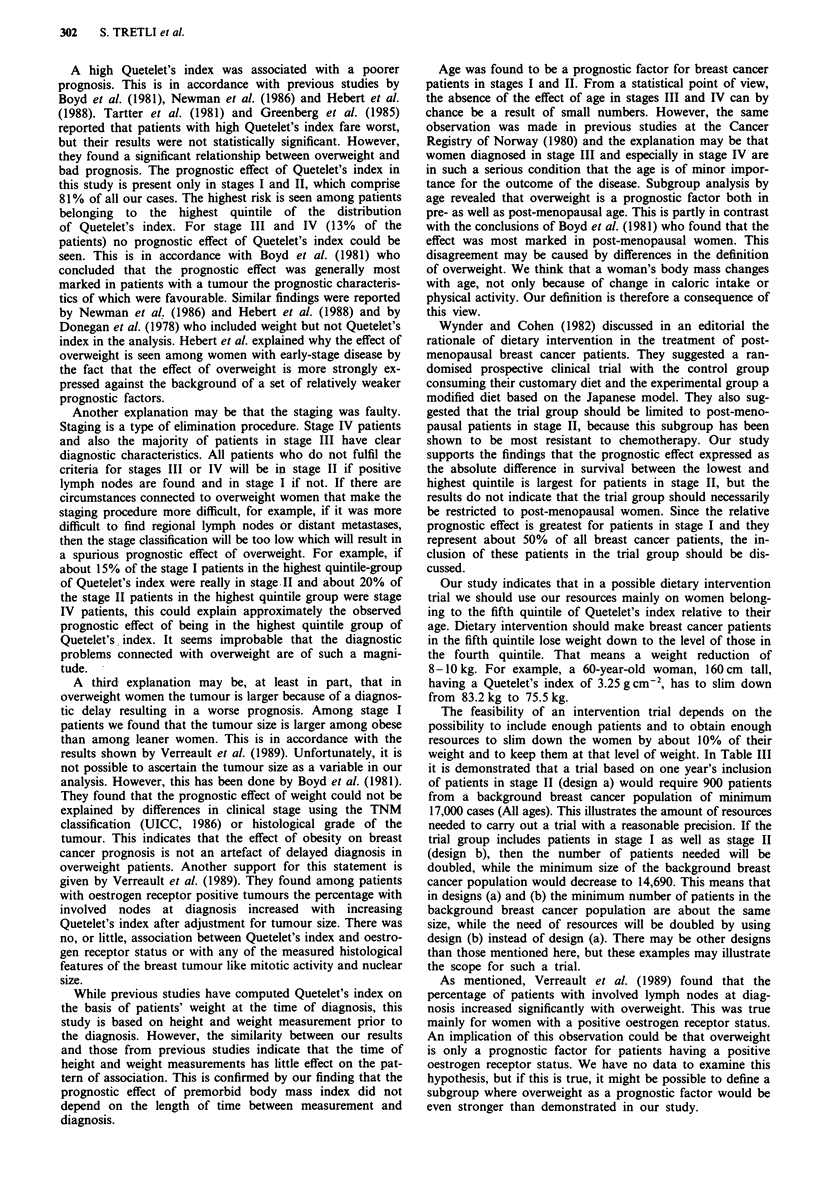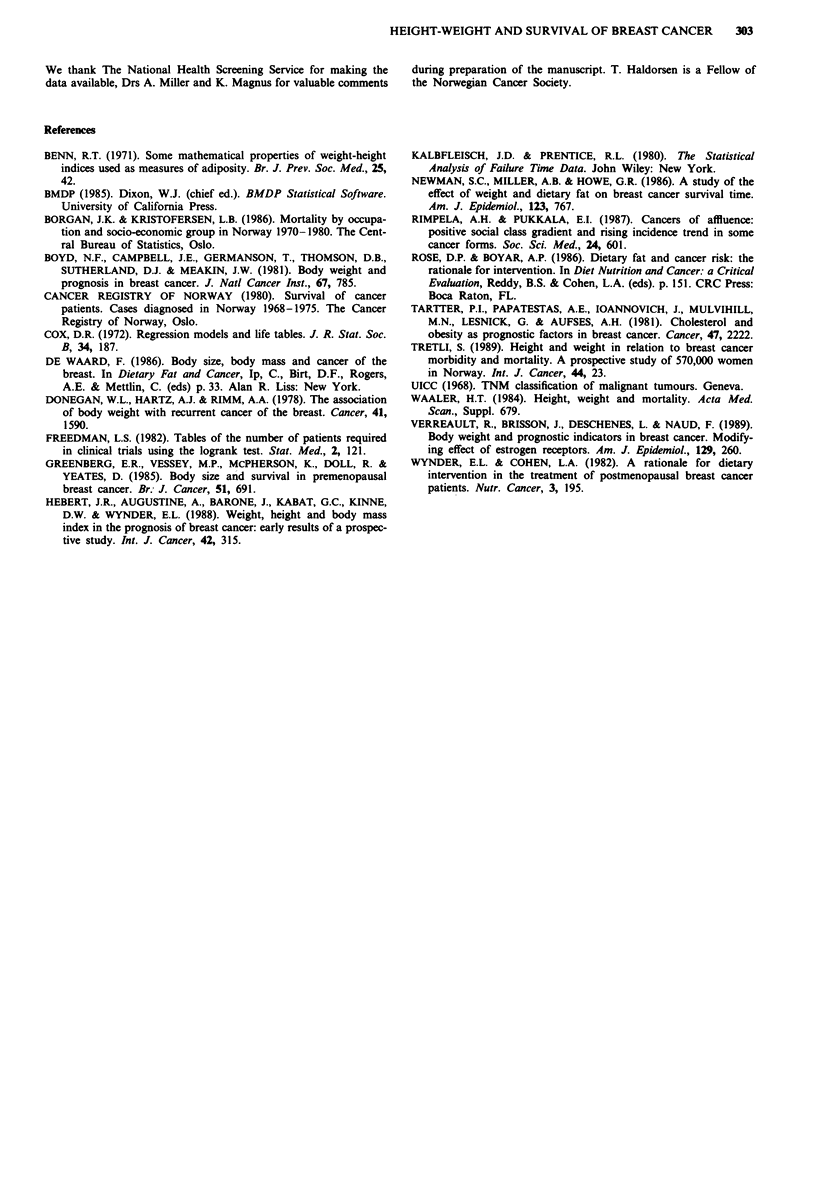# The effect of pre-morbid height and weight on the survival of breast cancer patients.

**DOI:** 10.1038/bjc.1990.282

**Published:** 1990-08

**Authors:** S. Tretli, T. Haldorsen, L. Ottestad

**Affiliations:** Cancer Registry of Norway, Institute of Epidemiological Cancer Research, Montebello, Oslo.

## Abstract

A total of 8,427 women with breast cancer with height and weight measured prior to the diagnosis were followed up for on average 4.3 years. 2,383 women died from breast cancer and 430 from other causes. Among women diagnosed without any metastasis (stage I) the death rate was 1.70 times higher for those belonging to the highest quintile of body mass with respect to age compared to those in the lowest quintile. For patients with involved lymph nodes at diagnosis (stage II) the death rate was 1.42 times higher. Overweight was not a prognostic factor for stages III and IV patients. The prognostic effect of body mass in stages I and II was mainly connected to those in the highest quintile and was found for women in pre- as well as post-menopausal age. The effect did not depend on the length of time between measurement and diagnosis. Height was not found to be of prognostic relevance. The idea of the feasibility of a dietary trial in terms of the minimum trial size is given.


Br. J. Cancer (1990), 62, 299–303 © Macmillan Press Ltd., 1990

### The effect of pre-morbid height and weight on the survival of breast cancer patients

S. Tretli[1], T. Haldorsen[1] & L. Ottestad[2]

[1]*The Cancer Registry of Norway, Institute of Epidemiological Cancer Research, Montebello, 0310 Oslo 3, Norway; and* [2]*The Norwegian Radium Hospital, Montebello, 0310 Oslo 3, Norway.*

**Summary** A total of 8,427 women with breast cancer with height and weight measured prior to the diagnosis were followed up for on average 4.3 years. 2,383 women died from breast cancer and 430 from other causes. Among women diagnosed without any metastasis (stage I) the death rate was 1.70 times higher for those belonging to the highest quintile of body mass with respect to age compared to those in the lowest quintile. For patients with involved lymph nodes at diagnosis (stage II) the death rate was 1.42 times higher. Overweight was not a prognostic factor for stages III and IV patients. The prognostic effect of body mass in stages I and II was mainly connected to those in the highest quintile and was found for women in pre- as well as post-menopausal age. The effect did not depend on the length of time between measurement and diagnosis. Height was not found to be of prognostic relevance. The idea of the feasibility of a dietary trial in terms of the minimum trial size is given.

In a recent study of the relation between height and weight and breast cancer morbidity (Tretli, 1989), height was found to be a risk factor independent of age. Overweight was a risk factor in the post-menopausal age-group while it appeared to protect against breast cancer in the pre-menopausal age-group. When stage I and stages II–IV were used as follow-up endpoints, a negative and positive association, respectively, with overweight was found indicating that overweight reflects enhanced growth. A reasonable extension of this study was to use the same series to look at premorbid height and weight as possible prognostic factors for the outcome of the disease. Most studies on the subject have shown that overweight aggravates the prognosis of breast cancer patients (Rose & Boyar, 1986). Height, however, has not been shown to be a prognostic factor (De Waard, 1986).

The size of our series is much greater than that of previous studies. This allows an analysis within each of the four stages of the disease. In this study we pay special attention to the fact that overweight ought to be considered relative to the person's age at time of measurement and we look for feasible weight groups with respect to possible dietary intervention trials.

### Materials and methods

Between 1963 and 1975 The National Mass Radiography Service measured the height and weight of all participants in a nationwide tuberculosis screening programme. The survey included all inhabitants over 15 years of age in 17 of 19 counties in Norway. The counties Oslo and Buskerud, which were not included, comprise 17.5% of the population of Norway. The study is based on this survey but restricted to the age-group 30–69 years at time of height and weight measurement. Altogether 567,333 women attended the screening in this age-group. The attendance rate was 85% with a range from 82% to 89% in the eight five-year age-groups. These women were followed until the end of 1981 through the population-based Norwegian Cancer Registry. 8,427 new cases of breast cancer were diagnosed and of these 97% were histologically verified. All these cases were followed up with respect to death in the same period by means of information from the Central Bureau of Statistics. The underlying cause of death on the death certificate was used. This linkage was made possible by the national system of personal identification numbers.

Correspondence: S. Tretli.
Received 22 September 1989; and in revised form 19 February 1990.

Height was measured to the nearest centimetre and weight to the nearest half kilogram on regularly calibrated scales. The persons were asked to undress the upper body because of the X-ray examination and to take off their shoes. A small number of measurements were made under special circumstances (e.g. pregnancy, kyphosis, wearing shoes etc.) and were therefore excluded.

The staging procedure which is used at the Cancer Registry of Norway, and thereby in this study, is based on clinical forms and histopathology reports. For nearly all breast cancer patients in this study, the breast was removed with varying degree of axillary lymph node toilette. Based on clinical as well as macro- and microscopical informations about extension of the primary tumour on the pathological forms, stage I, II or III was stated. Stage IV was stated if information from the clinical reports in a period of four months from the date of diagnosis, indicated distant metastases. The definition of stage at diagnosis used was: stage I, tumours of all sizes confined to the breast (except cases belonging to stage III); stage II, tumour in the breast with metastases to the axillary lymph nodes; stage III, tumour in the breast with direct extension to the skin or chest wall (may or may not have axillary lymph node metastases); stage IV, tumour in the breast with distant metastases.

Of a total of 8,427 breast cancer cases 4,019 cases (47.7%) were diagnosed in stage I, 2,808 cases (33.3%) in stage II, 464 cases (5.5%) in stage III and 636 cases (7.5%) in stage IV. For 500 cases the stage was unknown.

As a measure of obesity we have used Quetelet's index which is defined as weight divided by the height squared. This index has been shown to be strongly correlated to weight and almost uncorrelated to height (Benn, 1971). In our series weight divided by height raised to the power of 1.8 would have been uncorrelated to height. However, the change in results by using 1.8 instead of 2.0 as the exponent is so small that we have chosen to use Quetelet's index to make our results comparable to those of previous studies.

A special examination of the association between size of the tumour and Quetelet's index was carried out on a sample of patients treated at the Norwegian Radium Hospital. Fifty-five patients diagnosed in stage I belonging to the first quintile of Quetelet's index and 53 stage I patients in the fifth quintile were randomly selected. Information on the tumour size of these patients was collected from the hospital records where the TNM classification was used (UICC, 1968).

### *Statistical methods*

Cox's (1972) regression model is used in the survival analysis. Death from causes other than breast cancer was treated as censoring in the same manner as the end of follow-up period.

We felt that both height and Quetelet's index should be related to the woman's age, height because of a strong birth-cohort effect (Figure 1a) and Quetelet's index for biological reasons. The quintile groups for height and Quetelet's index were therefore defined within each of the 8 five-years age-groups at time of measurement for the total series of 567,333 women. The quintile-groups are hence specific to age as shown in Figure 1a,b.

The model used to estimate the intensity of death from breast cancer among the cases at time $t$ ($t$ denotes the time since diagnosis in months), is described by:

$$\lambda(t;\alpha_j,n_k,\theta_l) = \lambda_0(t)\ \exp(\alpha_j + n_k + \theta_l)\ j = 2,3;\ k = 2,..,5;\ 1 = 2,..,5$$

where $\lambda_0(t)$ is the baseline intensity for a patient who belongs to age-group 30–49 years at time of diagnosis, first quintile of relative height and first quintile of relative Quetelet's index. $\alpha_j$ is the age-effect categorised into 3 age-groups 30–49, 50–64, 65+ years, $n_k$ is the relative height-effect categorised into quintile groups and $\theta_l$ is the effect of Quetelet's index categorised into quintile groups. This means that we, for example, may think of $\exp(\alpha_3)$ as a relative risk due to the fact that a person's age is above 65 years at time of diagnosis instead of being 30–49 years.

The survival curve for a patient belonging to age-group $j$ at time of diagnosis, quintile-group $k$ of height and quintile-group $l$ of Quetelet's index, is given by:

$$S(t;\alpha_j,n_k,\theta_l) = \exp\ [-\int_0^t \lambda_0(s)ds * \exp(\alpha_j + n_k + \theta_l)]$$

$$j = 2,3;\ k = 2,..,5;\ l = 2,..,5.$$

The analysis is done within subgroups defined by the stage of the disease at time of diagnosis. The statistical package BMDP (1985) was used in the analysis. The adequacy of the proportional hazards assumptions in the regression models were checked by ln(-ln)-plots from stratified analysis for the variables included as covariates (Kalbfleisch & Prentice, 1980) and the assumptions were accepted.

The estimations of the sample size of a possible intervention trial are based on log rank statistics (Freedman, 1982) with a significance level of 0.05 and a statistical power of 0.80 of detecting a medically significant effect on the survival. In this case the effect corresponds to the difference between the survival rate of those in the fifth quintile and those in the fourth quintile of Quetelet's index. All computations are based on the women who belong to the fifth quintile of Quetelet's index and who are 30–75 years of age.

### Results

After an average follow-up period of 4.3 years (range: 0–18 years) 5,614 out of 8,427 breast cancer patients (66.6%) were still alive, 2,383 (28.3%) died from breast cancer and 430

**Figure 1** Age-specific limits for grouping height (**a**) and Quetelet's index (**b**) into quintiles (approximately) in the total series of 567,333 women. Q1, Q2, .., Q5 denotes the quintile number.

(5.1%) died from other diseases. The difference between 430 and the expected number of 507.8 based on death rates of the total population by age and year of birth, is statistically significant. Looking into subdiagnosis the differences between observed and expected number of deaths are small. The largest deviance is seen for 'other diseases than cancer and cardiovascular diseases' (65 deaths observed versus 95.7 expected).

Table I presents the results of Cox's regression analysis conducted within each of the four stages of the disease at time of diagnosis. The age at diagnosis is a prognostic factor for patients diagnosed in stages I or II but not for patients in stages III or IV.

Height is known to be a risk factor for breast cancer, but not a prognostic factor (De Waard, 1986). We found no prognostic effect of height in this study.

Quetelet's index appears to be a prognostic factor for breast cancer diagnosed in stage I or II of the disease but not for stages III and IV. Table I shows that the prognostic effect of the relative Quetelet's index is mainly connected to the highest quintile of the distribution. This means that there is a

**Table I** The relative risk of dying from breast cancer by categories of age at diagnosis, relative height and Quetelet's index within the four stages of breast cancer

| | | *Stage I Relative risk* | *Stage II Relative risk* | *Stage III Relative risk* | *Stage IV Relative risk* |
|---|---|---|---|---|---|
| Age at diagnosis (years) | 30–49 | 1.00 | 1.00 | 1.00 | 1.00 |
| | 50–64 | 1.32 (1.05, 1.66) | 1.32 (1.13, 1.53) | 1.05 (0.69, 1.59) | 1.21 (0.93, 1.57) |
| | 65+ | 1.73 (1.36, 2.21) | 1.41 (1.19, 1.68) | 1.07 (0.69, 1.66) | 1.15 (0.86, 1.52) |
| Relative height | 1.Quintile | 1.00 | 1.00 | 1.00 | 1.00 |
| | 2.Quintile | 1.03 (0.78, 1.35) | 0.97 (0.81, 1.17) | 0.78 (0.52, 1.19) | 0.96 (0.72, 1.27) |
| | 3.Quintile | 1.10 (0.84, 1.43) | 0.98 (0.81, 1.17) | 0.76 (0.50, 1.17) | 1.05 (0.81, 1.36) |
| | 4.Quintile | 0.89 (0.66, 1.20) | 0.96 (0.79, 1.17) | 0.93 (0.58, 1.49) | 0.95 (0.71, 1.27) |
| | 5.Quintile | 1.29 (0.99, 1.68) | 0.93 (0.77, 1.13) | 0.96 (0.62, 1.49) | 0.93 (0.71, 1.23) |
| Relative Quetelet's Index | 1.Quintile | 1.00 | 1.00 | 1.00 | 1.00 |
| | 2.Quintile | 1.17 (0.87, 1.56) | 1.12 (0.90, 1.38) | 0.85 (0.53, 1.35) | 0.88 (0.65, 1.20) |
| | 3.Quintile | 1.32 (1.00, 1.73) | 1.17 (0.96, 1.38) | 0.79 (0.51, 1.24) | 0.70 (0.52, 0.96) |
| | 4.Quintile | 1.38 (1.04, 1.83) | 1.10 (0.90, 1.35) | 0.94 (0.61, 1.43) | 0.89 (0.67, 1.18) |
| | 5.Quintile | 1.70 (1.29, 2.25) | 1.42 (1.17, 1.73) | 0.97 (0.63, 1.47) | 1.09 (0.82, 1.45) |

95% confidence intervals are given in parentheses. Study group: All breast cancer cases.

significant excess risk of dying if the Quetelet's index value in the age-group 30–34 years is above 2.55 g $cm^{-2}$ (for example: height = 160 cm and weight = 65.2 kg) and above 3.10 g $cm^{-2}$ (for example: height = 160 cm and weight = 79.4 kg) in the age-group 65–69 years. Although the relative risk of dying between highest and lowest quintile of Quetelet's index is higher in stage I than in stage II, the absolute prognostic effect is greater in stage II. The visualisation of this is seen in Figure 2a,b,c. The five year survival of patients in stage I is, in the three age-groups 30–49, 50–64 and above 65 years respectively 93%, 90% and 87% in the lowest quintile and 88%, 84% and 79% in the higest quintile. The corresponding figures for patients diagnosed in stage II are: 70%, 62% and 60% in the lowest quintile and 60%, 51% and 48% in the highest quintile.

The height and weight measurement is premorbid which in this connection means that the height and weight measurement was carried out before the breast cancer diagnosis. We have therefore examined whether the result shown in Table I was influenced by the time elapsed between height and weight measurement and diagnosis. This was done by running a sub-group analysis by dividing the time-span into three

**Figure 2** A comparison of survival rates of lowest (Q1) and highest (Q5) quintile of relative Quetelet's index within stage of breast cancer at diagnosis and by age-groups 30–49 years (**a**), 50–64 years (**b**), and 65 + years (**c**). The height parameter ($n_k$) = 1.00.

groups: 0–12 months, 13–59 months and 60 + months. The result (not demonstrated) shows no significant impact of this variable.

It has been claimed that the prognostic effect of overweight valid is for post-menopausal women only. A sub-group analysis by age does not confirm this point of view. The estimated prognostic effect of Quetelet's index was similar to the result shown in Table I for each of the three age-groups decribed in the model.

We also carried out a survival analysis when only the time interval from 24 months after diagnosis is taken into account. This implies that we are 'excluding' events in the active period of treatment. The result reveals that the prognostic effect of Quetelet's index in the fifth quintile becomes a little stronger (not shown).

Table II shows a significant positive association between tumour size and Quetelet's index ($P = 0.015$) obtained in the separate study. This study made it possible to check the quality of the Cancer Registry's staging of the patients (used in the main study) versus the clinical notes. The comparison reveals complete accordance.

We have made some computations to get an idea of the size of an intervention trial organised as a randomised two-group study among women belonging to the fifth quintile of the Quetelet's index. Table III shows the number needed and the minimim size of the background breast cancer population for four trial designs.

### Discussion

We found that death from other diseases was less frequent in breast cancer cases than in the general population when age and year of birth are taken into account. This may be explained by the fact that high socio-economic status is positively associated with breast cancer (Rimpela & Pukkala, 1987) and negatively associated with most other diseases than breast cancer (Borgan & Kristofersen, 1986). Overweight persons have a higher mortality in general and especially an excess mortality from cerebrovascular disease and diabetes (Waaler, 1984). The observed mortality from cerebrovascular disease and 'other disease' (including diabetes) was 137 versus 179.3 cases expected. It is unlikely that this difference, taking into account that 2,383 patients died from breast cancer, could significantly influence our results.

**Table II** Tumour size by extreme quintiles of Quetelet's index relative to age at height and weight measurement

| | *Tumour size*[a] | | | | |
|---|---|---|---|---|---|
| *Quetelet's index* | $T_0$ | $T_1$ | $T_2$ | $T_3$ | *Sum* |
| Quintile 1 | 0 | 42 | 13 | 0 | 55 |
| Quintile 5 | 2 | 26 | 23 | 2 | 53 |

[a]According to the TNM classification (UICC, 1968).

**Table III** Number of patients needed per year of inclusion and size of background breast cancer population by four different designs of an intervention trial

| *Design* | *Number of patients needed per year of inclusion* | *Size of the background breast cancer population* |
|---|---|---|
| (a) Stage II patients, 1 year of inclusion, 5 years of follow-up. | 900 | 17,000 |
| (b) Stage I and II patients, 1 year of inclusion, 5 years of follow-up. | 1,880 | 14,690 |
| (c) Stage II patients, 3 years of inclusion, 3–5 years of follow-up | 365 | 6,800 |
| (d) Stage I and II patients, 3 year of inclusion, 3–5 years of follow-up. | 750 | 5,860 |

A high Quetelet's index was associated with a poorer prognosis. This is in accordance with previous studies by Boyd et al. (1981), Newman et al. (1986) and Hebert et al. (1988). Tartter et al. (1981) and Greenberg et al. (1985) reported that patients with high Quetelet's index fare worst, but their results were not statistically significant. However, they found a significant relationship between overweight and bad prognosis. The prognostic effect of Quetelet's index in this study is present only in stages I and II, which comprise 81% of all our cases. The highest risk is seen among patients belonging to the highest quintile of the distribution of Quetelet's index. For stage III and IV (13% of the patients) no prognostic effect of Quetelet's index could be seen. This is in accordance with Boyd et al. (1981) who concluded that the prognostic effect was generally most marked in patients with a tumour the prognostic characteristics of which were favourable. Similar findings were reported by Newman et al. (1986) and Hebert et al. (1988) and by Donegan et al. (1978) who included weight but not Quetelet's index in the analysis. Hebert et al. explained why the effect of overweight is seen among women with early-stage disease by the fact that the effect of overweight is more strongly expressed against the background of a set of relatively weaker prognostic factors.

Another explanation may be that the staging was faulty. Staging is a type of elimination procedure. Stage IV patients and also the majority of patients in stage III have clear diagnostic characteristics. All patients who do not fulfil the criteria for stages III or IV will be in stage II if positive lymph nodes are found and in stage I if not. If there are circumstances connected to overweight women that make the staging procedure more difficult, for example, if it was more difficult to find regional lymph nodes or distant metastases, then the stage classification will be too low which will result in a spurious prognostic effect of overweight. For example, if about 15% of the stage I patients in the highest quintile-group of Quetelet's index were really in stage II and about 20% of the stage II patients in the highest quintile group were stage IV patients, this could explain approximately the observed prognostic effect of being in the highest quintile group of Quetelet's index. It seems improbable that the diagnostic problems connected with overweight are of such a magnitude.

A third explanation may be, at least in part, that in overweight women the tumour is larger because of a diagnostic delay resulting in a worse prognosis. Among stage I patients we found that the tumour size is larger among obese than among leaner women. This is in accordance with the results shown by Verreault et al. (1989). Unfortunately, it is not possible to ascertain the tumour size as a variable in our analysis. However, this has been done by Boyd et al. (1981). They found that the prognostic effect of weight could not be explained by differences in clinical stage using the TNM classification (UICC, 1986) or histological grade of the tumour. This indicates that the effect of obesity on breast cancer prognosis is not an artefact of delayed diagnosis in overweight patients. Another support for this statement is given by Verreault et al. (1989). They found among patients with oestrogen receptor positive tumours the percentage with involved nodes at diagnosis increased with increasing Quetelet's index after adjustment for tumour size. There was no, or little, association between Quetelet's index and oestrogen receptor status or with any of the measured histological features of the breast tumour like mitotic activity and nuclear size.

While previous studies have computed Quetelet's index on the basis of patients' weight at the time of diagnosis, this study is based on height and weight measurement prior to the diagnosis. However, the similarity between our results and those from previous studies indicate that the time of height and weight measurements has little effect on the pattern of association. This is confirmed by our finding that the prognostic effect of premorbid body mass index did not depend on the length of time between measurement and diagnosis.

Age was found to be a prognostic factor for breast cancer patients in stages I and II. From a statistical point of view, the absence of the effect of age in stages III and IV can by chance be a result of small numbers. However, the same observation was made in previous studies at the Cancer Registry of Norway (1980) and the explanation may be that women diagnosed in stage III and especially in stage IV are in such a serious condition that the age is of minor importance for the outcome of the disease. Subgroup analysis by age revealed that overweight is a prognostic factor both in pre- as well as post-menopausal age. This is partly in contrast with the conclusions of Boyd et al. (1981) who found that the effect was most marked in post-menopausal women. This disagreement may be caused by differences in the definition of overweight. We think that a woman's body mass changes with age, not only because of change in caloric intake or physical activity. Our definition is therefore a consequence of this view.

Wynder and Cohen (1982) discussed in an editorial the rationale of dietary intervention in the treatment of post-menopausal breast cancer patients. They suggested a randomised prospective clinical trial with the control group consuming their customary diet and the experimental group a modified diet based on the Japanese model. They also suggested that the trial group should be limited to post-menopausal patients in stage II, because this subgroup has been shown to be most resistant to chemotherapy. Our study supports the findings that the prognostic effect expressed as the absolute difference in survival between the lowest and highest quintile is largest for patients in stage II, but the results do not indicate that the trial group should necessarily be restricted to post-menopausal women. Since the relative prognostic effect is greatest for patients in stage I and they represent about 50% of all breast cancer patients, the inclusion of these patients in the trial group should be discussed.

Our study indicates that in a possible dietary intervention trial we should use our resources mainly on women belonging to the fifth quintile of Quetelet's index relative to their age. Dietary intervention should make breast cancer patients in the fifth quintile lose weight down to the level of those in the fourth quintile. That means a weight reduction of 8–10 kg. For example, a 60-year-old woman, 160 cm tall, having a Quetelet's index of $3.25 \text{ g cm}^{-2}$, has to slim down from 83.2 kg to 75.5 kg.

The feasibility of an intervention trial depends on the possibility to include enough patients and to obtain enough resources to slim down the women by about 10% of their weight and to keep them at that level of weight. In Table III it is demonstrated that a trial based on one year's inclusion of patients in stage II (design a) would require 900 patients from a background breast cancer population of minimum 17,000 cases (All ages). This illustrates the amount of resources needed to carry out a trial with a reasonable precision. If the trial group includes patients in stage I as well as stage II (design b), then the number of patients needed will be doubled, while the minimum size of the background breast cancer population would decrease to 14,690. This means that in designs (a) and (b) the minimum number of patients in the background breast cancer population are about the same size, while the need of resources will be doubled by using design (b) instead of design (a). There may be other designs than those mentioned here, but these examples may illustrate the scope for such a trial.

As mentioned, Verreault et al. (1989) found that the percentage of patients with involved lymph nodes at diagnosis increased significantly with overweight. This was true mainly for women with a positive oestrogen receptor status. An implication of this observation could be that overweight is only a prognostic factor for patients having a positive oestrogen receptor status. We have no data to examine this hypothesis, but if this is true, it might be possible to define a subgroup where overweight as a prognostic factor would be even stronger than demonstrated in our study.

We thank The National Health Screening Service for making the data available, Drs A. Miller and K. Magnus for valuable comments during preparation of the manuscript. T. Haldorsen is a Fellow of the Norwegian Cancer Society.

### References

BENN, R.T. (1971). Some mathematical properties of weight-height indices used as measures of adiposity. *Br. J. Prev. Soc. Med.*, **25**, 42.

BMDP (1985). Dixon, W.J. (chief ed.). *BMDP Statistical Software*. University of California Press.

BORGAN, J.K. & KRISTOFERSEN, L.B. (1986). Mortality by occupation and socio-economic group in Norway 1970–1980. The Central Bureau of Statistics, Oslo.

BOYD, N.F., CAMPBELL, J.E., GERMANSON, T., THOMSON, D.B., SUTHERLAND, D.J. & MEAKIN, J.W. (1981). Body weight and prognosis in breast cancer. *J. Natl Cancer Inst.*, **67**, 785.

CANCER REGISTRY OF NORWAY (1980). Survival of cancer patients. Cases diagnosed in Norway 1968–1975. The Cancer Registry of Norway, Oslo.

COX, D.R. (1972). Regression models and life tables. *J. R. Stat. Soc. B*, **34**, 187.

DE WAARD, F. (1986). Body size, body mass and cancer of the breast. In *Dietary Fat and Cancer*, Ip, C., Birt, D.F., Rogers, A.E. & Mettlin, C. (eds) p. 33. Alan R. Liss: New York.

DONEGAN, W.L., HARTZ, A.J. & RIMM, A.A. (1978). The association of body weight with recurrent cancer of the breast. *Cancer*, **41**, 1590.

FREEDMAN, L.S. (1982). Tables of the number of patients required in clinical trials using the logrank test. *Stat. Med.*, **2**, 121.

GREENBERG, E.R., VESSEY, M.P., MCPHERSON, K., DOLL, R. & YEATES, D. (1985). Body size and survival in premenopausal breast cancer. *Br. J. Cancer*, **51**, 691.

HEBERT, J.R., AUGUSTINE, A., BARONE, J., KABAT, G.C., KINNE, D.W. & WYNDER, E.L. (1988). Weight, height and body mass index in the prognosis of breast cancer: early results of a prospective study. *Int. J. Cancer*, **42**, 315.

KALBFLEISCH, J.D. & PRENTICE, R.L. (1980). *The Statistical Analysis of Failure Time Data*. John Wiley: New York.

NEWMAN, S.C., MILLER, A.B. & HOWE, G.R. (1986). A study of the effect of weight and dietary fat on breast cancer survival time. *Am. J. Epidemiol.*, **123**, 767.

RIMPELA, A.H. & PUKKALA, E.I. (1987). Cancers of affluence: positive social class gradient and rising incidence trend in some cancer forms. *Soc. Sci. Med.*, **24**, 601.

ROSE, D.P. & BOYAR, A.P. (1986). Dietary fat and cancer risk: the rationale for intervention. In *Diet Nutrition and Cancer: a Critical Evaluation*, Reddy, B.S. & Cohen, L.A. (eds). p. 151. CRC Press: Boca Raton, FL.

TARTTER, P.I., PAPATESTAS, A.E., IOANNOVICH, J., MULVIHILL, M.N., LESNICK, G. & AUFSES, A.H. (1981). Cholesterol and obesity as prognostic factors in breast cancer. *Cancer*, **47**, 2222.

TRETLI, S. (1989). Height and weight in relation to breast cancer morbidity and mortality. A prospective study of 570,000 women in Norway. *Int. J. Cancer*, **44**, 23.

UICC (1968). TNM classification of malignant tumours. Geneva.

WAALER, H.T. (1984). Height, weight and mortality. *Acta Med. Scan.*, Suppl. 679.

VERREAULT, R., BRISSON, J., DESCHENES, L. & NAUD, F. (1989). Body weight and prognostic indicators in breast cancer. Modifying effect of estrogen receptors. *Am. J. Epidemiol.*, **129**, 260.

WYNDER, E.L. & COHEN, L.A. (1982). A rationale for dietary intervention in the treatment of postmenopausal breast cancer patients. *Nutr. Cancer*, **3**, 195.